# High Rates of Germline Pathogenic Variants in Somali Patients with Ovarian Cancer

**DOI:** 10.1016/j.gore.2024.101538

**Published:** 2024-10-29

**Authors:** José V. Somohano-Short, Natasha Crawford, Mahmoud A. Khalifa, Britt K. Erickson

**Affiliations:** a Dept. of Obstetrics, Gynecology, and Women’s Health – University of Minnesota, Minneapolis, MN, USA; b Division of Gynecologic Oncology– University of Minnesota, Minneapolis, MN, USA; c Dept. of Laboratory Medicine and Pathology – University of Minnesota, Minneapolis, MN, USA; d Ponce Health Sciences University School of Medicine, Ponce, PR, USA; e University of Minnesota College of Medicine, Duluth, MN, USA

**Keywords:** BRIP1 pathogenic variant, Ovarian cancer, Somali women, Germline testing, Healthcare disparities, Genetic screening

## Abstract

The objective of this study was to determine the rate of germline high risk ovarian cancer susceptibility pathogenic variants in Somali patients with ovarian carcinoma treated at a single institution between 2015 and 2022. Out of eight identified patients, five underwent germline and/or somatic testing, revealing a high prevalence (3 of 5, 60 %) of a BRIP1 splice site mutation (c.1936 + 1G > A). Additionally, one patient had a BRCA2 pathogenic variant, and two had the same MLH1 variant of uncertain significance. The high prevalence of BRIP1 pathogenic variants warrants further study into a possible founder effect within the Somali population, emphasizing the need for targeted genetic screening and counseling. The study also highlights significant barriers to genetic testing, pointing to the critical role of healthcare disparities and social determinants of health (SDoH) in cancer outcomes. Comprehensive genomic profiling and community-based research are essential to address these disparities and improve cancer care for this underserved population. Larger studies are needed to validate these findings and to develop tailored interventions that enhance the prevention, diagnosis, treatment, and prognosis of ovarian cancer in Somali women.

## 1 Background

The frequency of hereditary *BRCA1/BRCA2* pathogenic variants in patients with ovarian cancer (OC) is between 15–18 %. However, up to 4 % of patients have other germline pathogenic variants which put them at risk for this disease for example including other variants in homologous recombination deficiency (Norquist et al., 2016). *BRIP1* (*BRCA1* Interacting Protein) is a hereditary breast and ovarian cancer (HBOC) associated gene that encodes for a DNA helicase that works in conjunction with *BRCA1* in homologous recombination DNA repair (Seal et al., 2006). *BRIP1* pathogenic variants are associated with up to an 8-fold increased risk of ovarian and fallopian tube cancers (Torre et al., 2018). When compared to other demographic groups, non-Hispanic Black patients report the lowest incidence of ovarian cancers. Alarmingly, however, this same group has the lowest 5-year survival rate for all epithelial cancer stages and ovarian cancer subtypes combined (Seal et al., 2006). Healthcare disparities and social determinants of health (SDoH) account for many of these outcome disparities; some studies report complete resolution of outcome differences when data sets are adjusted to account for SDoH (O'Donnell et al., 2018). Given the relative subjective nature of how study subjects’ demographics are classified and the highly-complex task of quantifying SDoH, it is difficult to assess whether these data are mirrored in all Black patients. We hypothesize that, in addition to healthcare disparities and SDoH, highly specific patient populations share unique cancer characteristics that could contribute to the observed overall survival rates. The state of Minnesota harbors over 78,000 Somali immigrants, the largest Somali population in the United States and we therefore sought to determine more detailed information on germline pathogenic variants (Bundy et al., 1999).

## 2 Objective

To determine the rate of pathogenic germline pathogenic variants in a cohort of Somali women with ovarian/fallopian tube/primary peritoneal carcinoma.

## 3 Methods

We conducted a retrospective case-series evaluating all ovarian cancer patients who identified as Somalian that presented to University of Minnesota’s Division of Gynecologic Oncology between 2015 and 2022. IRB approval was obtained. Patients with ovarian cancer were identified through ICD-10 coding as well as through a surgical pathology database. Somali patients were identified by a combination of the electronic medical record’s reported country of origin, language preferences, social history and other clinical notes. Clinical, pathologic, treatment, and survival data were abstracted from the medical record. All tumor testing and germline testing were performed through commercial testing platforms and available as part of the medical record.

## 4 Results

We identified 8 patients of Somali ethnicity with epithelial ovarian/fallopian tube/primary peritoneal carcinoma, all of whom met germline testing criteria as recommended by the National Comprehensive Cancer Center Network (Somali population) (Table 1). Six patients had advanced stage high grade serous carcinoma, one patient had low grade serous carcinoma, and one patient had early stage high grade serous carcinoma. Median age was 65 (range 48–79). All patients received platinum and taxane based chemotherapy. Three of 8 received PARP inhibitor therapy with varying degrees of adherence.

**Table 1:** High Rates of High-Grade Serous Histology and Germline Pathogenic Variants.

**#**	**Age**	**FIGO Stage**	**Histology**	**Germline Testing**	**VUS**	**Somatic Testing**	**PARPi**
1	69	IVA	High-Grade Serous	**BRIP1 c.1936-1G > A**	ATM c.7808A > G, BRCA2 c.5808G > A, CDH1 c.2329G > A, CHEK2 c.8G > T,	BRIP c.1936-1G > A, TP53 V122fs*46	Yes − On Maintenance
2	65	IIIC	High-Grade Serous	**BRIP1 c.1936-1G > A**	MLH1 c.1117G > A	Not performed or available	Yes, one month only − Discontinued
3	51	IIIC	High-Grade Serous	Not performed	Not performed	**BRIP c.1936-1G > A, TP53 Y23C**	No
4	77	IV	High-Grade Serous	**BRCA2 c.1707_1708delGA**	MLH1 c.1117G > A	Not performed or available	Yes − Discontinued after 4 months (Hip Fx + thrombocytopenia)
5	48	IIB	Low-Grade Serous	No significant mutations detected	DICER1 c.3462A > C	Not performed or available	No
6	65	IVA	High grade serous	No mutations identified	No mutations identified	Not performed	No
7	63	IVB	High-Grade Serous	Not performed	Not performed	Not performed	No
8	79	IC1	High grade serous	Not performed	Not performed	Not performed	No

Five of 8 patients (62.5 %) underwent germline testing. Two of 8 patients (25 %) underwent comprehensive somatic tumor testing (including 1 who did not undergo germline testing). Three of 8 patients (2 by germline testing and 1 by somatic testing only) had the same *BRIP1* splice site c.1936 + 1G > A pathogenic variant. One patient carried a *BRCA2* germline pathogenic variant. Two patients harbored the same germline VUS (*MLH1* c.1117G > A). None of the patients are known to be related. Specifically, medical history was reviewed from the gynecologic oncology initial patient interview as well as the more detailed visit with the genetic counselor. We could not identify any clear familial connections based on this review. (Table 1).

## 5 Discussion

This small case series represents a preliminary investigation into the genetic landscape of ovarian cancer in a specific population of Somali women in Minnesota, who are one of the largest African immigrant communities in the United States. Our findings provide insight that could inform both clinical management and future research directions. The identification of specific genetic pathogenic variants in this cohort underscores the importance of understanding the unique genetic profiles of different ethnic groups, which could significantly impact diagnosis, treatment, and prognosis of ovarian cancer.

This study revealed a high prevalence of a *BRIP1* splice site pathogenic variant (c.1936 + 1G > A) among Somali women with ovarian cancer, with 60 % of those tested (3 out of 5) carrying this pathogenic variant. This, of course, is notably higher than what is typically observed in other populations (Norquist et al., 2016, Seal et al., 2006). While our data is insufficient to conclude a founder effect, the patter warrants further investigation into the possibility of high rates of *BRIP1* pathogenic variants in the Somali Diaspora. This study suggests Somali women may benefit from targeted genetic screening and counseling. Additionally, the presence of a *BRCA2* pathogenic variant and recurrent variants of uncertain significance (VUS) in the *MLH1* gene further emphasize the diverse genetic landscape and the necessity for comprehensive genetic testing.

Clinically, the majority of patients in this study presented with advanced-stage high-grade serous carcinoma, which aligns with the stage at diagnosis for most ovarian cancer patients. The median age of 65 years at diagnosis is also consistent with the typical age range for ovarian cancer diagnosis. All patients received standard-of-care platinum and taxane-based chemotherapy and 3 of 8 patients were treated with PARP inhibitors. Notably however, not all patients received standard of care germline testing and tumor testing. Healthcare disparities and social determinants of health play a crucial role in cancer outcomes. The Somali immigrant community may face unique challenges, such as language barriers, cultural differences, discrimination from the health care system (including provider biases), and limited access to healthcare services, which can impede timely diagnosis and treatment. This highlights the need for community based research focused on understanding and addressing the Somali immigrants’ barriers to cancer care.

The limitations of this study include a small sample size and lack of comparative data, highlighting the need for larger, more comprehensive research to validate these findings. Because of the retrospective nature of this study, family history could not be corroborated directly with the patients. Moreover, we have limited data to understand the best methods for history-taking in a culturally appropriate and sensitive manner. Understanding the potential founder effects and historical context of Somali immigrants could provide further insights into the genetic predispositions observed.

This study provides a critical first step in understanding the genetic landscape of ovarian cancer in Somali women in Minnesota. The high prevalence of germline pathogenic variants and the presence of other significant genetic variations underscore the need for personalized treatment approaches and comprehensive genetic testing. Addressing healthcare disparities and considering the socio-economic context of the Somali immigrant community are vital for improving cancer outcomes. Continued research and tailored interventions are essential to provide equitable and effective cancer care for this underserved population.

## 6 Conclusions

In this small case series of Somali women with epithelial ovarian cancer, not all patients received standard-of-care germline testing. Rates of somatic testing were also lower than expected. A higher than expected rate of BRIP1 pathogenic variants were identified, suggesting the possibility of an unreported founder pathogenic variant relevant to the Somali diaspora. However, larger numbers are needed to verify these findings. These findings highlight the need to evaluate specific ethnic cancer risk assessments beyond the traditional categories of race.

**Author contributions**

The authors of this manuscript confirm contributions as follows:

Study conception and design: José V. Somohano-Short and Britt K. Erickson

Manuscript preparation: José V. Somohano-Short and Natasha Crawford.

Data collection: José V. Somohano-Short, Natasha Crawford, Mahmoud A. Khalifa, and Britt K. Erickson

Analysis and interpretation of results: José V. Somohano-Short and Britt K. Erickson

All Authors reviewed and approved the final version of this manuscript.

**Consent**: Written informed consent was obtained from the patient for publication of this case report and accompanying images.

## CRediT authorship contribution statement

**José V. Somohano-Short:** Writing – review & editing, Writing – original draft, Methodology, Investigation, Funding acquisition. **Natasha Crawford:** Writing – review & editing, Data curation. **Mahmoud A. Khalifa:** Investigation, Formal analysis, Data curation. **Britt K. Erickson:** Writing – review & editing, Writing – original draft, Supervision, Methodology, Investigation, Formal analysis, Conceptualization.

## Declaration of competing interest

The authors declare that they have no known competing financial interests or personal relationships that could have appeared to influence the work reported in this paper.
